# Targeting Gastrointestinal Cancers with Carvacrol: Mechanistic Insights and Therapeutic Potential

**DOI:** 10.3390/biom15060777

**Published:** 2025-05-27

**Authors:** Nitika Patwa, Gagandeep Singh, Vikas Sharma, Priyanka Chaudhary, Bunty Sharma, Shafiul Haque, Vikas Yadav, Shakti Ranjan Satapathy, Hardeep Singh Tuli

**Affiliations:** 1 Department of Biotechnology, DAV College, Ambala 133001, Haryana, India; nitika0491@gmail.com; 2 Section of Microbiology , Central Ayurveda Research Institute, Jhansi 284003, Uttar Pradesh, India; gsk.ccras@gmail.com; 3 Department of Biotechnology, Ambala College of Engineering and Applied Research, Ambala 133001, Haryana, India; vikas.bt@ambalacollege.ac.in; 4 Department of Zoology, DAV College (Lahore), Ambala 133001, Haryana, India; priyankadavambala@gmail.com; 5 Department of Biotechnology, Graphic Era (Deemed to Be University), Dehradun 248001, Uttarakhand, India; sharmabunty097@gmail.com; 6 Department of Nursing, College of Nursing and Health Sciences, Jazan University, Jazan 45142, Saudi Arabia; shafiul.haque@hotmail.com; 7 School of Medicine, Universidad Espiritu Santo, Samborondon 091650, Ecuador; 8 Cell & Experimental Pathology, Department of Translational Medicine, Lund University, 200 41 Malmo, Sweden; vikas.yadav@med.lu.se; 9 Department of Bio-Sciences and Technology, Maharishi Markandeshwar Engineering College, Maharishi Markandeshwar (Deemed to Be University), Mullana, Ambala 133001, Haryana, India

**Keywords:** carvacrol, gastrointestinal cancers, anticancer mechanisms, nanotechnology, synergistic therapy

## Abstract

Gastrointestinal (GI) cancers, including esophageal, gastric, pancreatic, liver, and colorectal malignancies, represent a major global health burden due to their high incidence, aggressive nature, and limited treatment outcomes. This review explores the therapeutic potential of carvacrol, a naturally occurring monoterpenoid phenol predominantly found in oregano and other aromatic plants. Carvacrol has demonstrated strong anticancer properties by modulating multiple molecular pathways governing apoptosis, inflammation, angiogenesis, and metastasis. Preclinical studies have revealed its ability to selectively target cancer cells while sparing healthy tissue. Advances in nanotechnology have further enhanced its pharmacological profile by improving solubility, stability, and tumor-targeted delivery. Additionally, carvacrol shows synergistic effects when used in combination with conventional chemotherapeutics. While the evidence is promising, clinical studies are needed to validate its translational potential. This review aims to consolidate current findings and encourage further investigation into carvacrol’s application as an adjunct or alternative therapeutic agent in GI cancer management.

## 1. Introduction

Cancer remains one of the most pressing global health challenges due to its multifactorial nature, high metastatic potential, and steadily increasing prevalence and mortality [1]. It is the second leading cause of death worldwide after cardiovascular diseases, with gastrointestinal (GI) cancers alone accounting for approximately 25% of all new cancer cases and more than one-third of cancer-related deaths. Globally, it is responsible for ten million deaths in 2020, or nearly one in every six deaths, surpassing the combined fatalities from AIDS, tuberculosis, and malaria [2]. Factors contributing to GI cancer development include genetic predispositions, viral infections, chronic inflammation, and lifestyle-related risks such as tobacco use, alcohol consumption, poor diet, and exposure to environmental toxins [3]. While chemotherapy remains a mainstay in cancer treatment, it is often accompanied by severe toxicities that limit its long-term use and diminish patients’ quality of life [4,5,6]. For instance, anthracyclines, including doxorubicin, are associated with cardiac toxicity, potentially leading to heart failure and cardiomyopathy [7]; Bleomycin can cause pulmonary toxicity, increasing the risk of lung fibrosis and respiratory failure; Cyclophosphamide induces bladder toxicity, resulting in hemorrhagic cystitis and bladder inflammation [8,7,9,10]. These challenges underscore the urgent need for safer, more effective therapeutic alternatives.

As researchers look for more effective cancer treatments with fewer side effects, natural products are receiving more scientific interest as potential chemopreventive agents [11,12]. By focusing on important biochemical pathways involved in tumor genesis and progression, phytochemicals produced from medicinal plants have shown promise in reducing the risk of cancer [13]. They are a promising substitute for synthetic pharmaceuticals due to their increased bioavailability, safety, effectiveness, and accessibility. These substances interact with many cellular functions to show anticancer potential, making them promising treatment possibilities. These natural compounds have anticancer effects by inhibiting the epithelial-to-mesenchymal transition, causing cell cycle arrest, promoting apoptosis, downregulating anti-apoptotic proteins, modulating signaling pathways, preventing epigenetic changes, and lowering DNA damage caused by oxidative stress. Their multi-pronged approach minimizes side effects typically associated with traditional chemotherapy while highlighting their significance in cancer treatment and prevention [14,15].

Because of its strong perfume and distinct flavor, oregano (*Origanum vulgare*), a hardy perennial herb native to Europe and Central Asia, is used in various recipes, such as salads, pizzas, and sausages. In addition to its culinary relevance, oregano essential oil (OEO) has garnered significant attention in food science and biological research due to its numerous bioactive properties. It is a highly prized natural substance because of its antibacterial, antioxidant, anti-inflammatory, and anticancer qualities. About 84.38% of OEO’s contents are ‘carvacrol’. This naturally occurring phenolic compound has been thoroughly investigated for its diverse biological effects because of its capacity to alter important molecular targets implicated in cancer development [16]. This broad spectrum of biological activity has led to its extensive investigation. Carvacrol has been demonstrated in studies to limit metastasis, induce apoptosis, suppress tumor cell proliferation, and improve the effectiveness of traditional chemotherapy medications [17]. Numerous studies have examined carvacrol’s anti-proliferative and anticancer qualities, demonstrating its promise as a natural medicine [18]. Its therapeutic potential is further increased by its capacity to specifically target cancer cells while preserving healthy cells. This review summarizes the key findings on carvacrol’s anticancer properties and offers useful information to direct future studies.

Given carvacrol’s broad therapeutic relevance across multiple cancer types, a focused review of its mechanisms in GI cancers is timely and warranted. Therefore, we conducted a comprehensive literature review to evaluate the preclinical evidence and potential translational applications.

## 2. Methodology

A comprehensive literature search was conducted using PubMed, Scopus, and Google Scholar to identify relevant studies on the anticancer properties of carvacrol. This review included peer-reviewed, full-length articles published between August 2004 and January 2025. The following inclusion and exclusion criteria guided the selection process.

 Inclusion Criteria:

• Studies investigating the effects of carvacrol on esophageal, gastric, colorectal, pancreatic, and liver cancer using animal models or in vitro experiments.
• Research evaluating carvacrol alone or in combination with other bioactive compounds.
• Preclinical studies (both in vitro and in vivo) that report on molecular alterations (such as altered gene or protein expression) or biological outcomes (such as inhibiting tumor growth, halting metastasis, or inducing apoptosis).
• English-language publications in peer-reviewed journals.

Exclusion Criteria:

• Research studies that do not specifically focus on the cancers of interest (e.g., general cancer research or studies on mixed cancer types without separate analyses).
• Research that does not evaluate the effects of carvacrol.
• Articles missing applicable biological, clinical, or molecular results or statistics.
• Non-peer-reviewed publications.
• Non-English-language publications.

## 3. Chemistry and Derivatization

To understand the basis of its biological activity, a foundational understanding of carvacrol’s chemical structure and derivatives is essential. Carvacrol (C_10_H_14_O), also known as 2-methyl-5-isopropylphenol, is a phenolic monoterpenoid having a molecular weight of 150.22 g/mol (CAS 499-75-2). At room temperature (25 °C), it is a thick, colorless to light yellow liquid with a strong, spicy smell and a relative density of 0.976 g/mL. Carvacrol has lipophilic properties, meaning it is highly soluble in ethanol and acetone but insoluble in water. Its melting and boiling temperatures are 1 °C and 237–238 °C, respectively, and its refractive index falls between 1.5210 and 1.5260 [19,20,21]. Carvacrol is naturally present in the essential oils (EOs) of various aromatic plants, including oregano (*Origanum vulgare*), thyme (*Thymus vulgaris*), Shirazi thyme (*Zataria multiflora Boiss*), ajwain (*Carum copticum*), pepperwort (*Lepidium flavum*), black cumin (*Nigella sativa* L.), and wild bergamot (*Citrus aurantium var. bergamia Loisel*) [22]. Carvacrol shows biological activities, such as antimicrobial, antitumor, antimutagenic, antigenotoxic, anti-inflammatory, anti-angiogenic, hepatoprotective, and antihepatotoxic properties. Because of the hydroxyl groups on its phenolic ring, carvacrol reduces oxidative damage and improves biological stability by giving hydrogen to peroxy radicals during lipid oxidation [23]. The anticancer properties of carvacrol have been mentioned in preclinical models of breast, liver, and lung carcinomas, appearing in the pro-apoptotic process [20,23]. Alamri and colleagues demonstrated that changes such as substituting ethers, esters, and acetic acid groups for the acidic proton enhance the molecule’s deadly effect on HeLa cells [24]. Carvacrol-derived copper–Schiff base complex prevented A549 lung cancer cells from proliferating and migrating during the G2/M cell cycle phase. Importantly, these compounds maintained above 50% viability in healthy cells, suggesting they are safe [25]. Additionally, in silico studies on carvacrol–aldehyde hybrids indicated anti-metastatic properties [26]. Similarly, Morita–Baylis–Hillman adducts derived from carvacrol have shown potential as anticancer agents [27]. The other carvacrol derivative, 4,4′-methylene bis (5-isopropyl-2-methyl) phenol, exhibits strong antioxidant activity with low toxicity, making it a promising candidate for cancer treatment [28].

## 4. Pathophysiology of Gastrointestinal Cancer

Various genetic alterations and perturbed signaling pathways cause gastrointestinal (GI) malignancies to grow, invade, and spread uncontrollably by interfering with normal cell cycle regulation. Cyclin D1 is produced in more significant amounts when the *CCND1* gene is amplified in esophageal cancer, which promotes tumor growth by accelerating cell division [29]. Furthermore, dysregulation of *RB1*, whether through loss or mutation, leads to overexpression of p16 as a compensatory mechanism. Nevertheless, without functional *RB1*, cells persist in dividing uncontrollably, which worsens the prognosis [30]. The overexpression or amplification of *HER2* further facilitates aggressive tumor behavior, while the EGFR activation enhances tumor proliferation and survival [31,32,33]. Changes in the *CDH1* gene, which produces E-Cadherin, cause cell adhesion to be lost in gastric cancer, which increases the tumor’s aggressiveness and potential for metastasis, particularly in hereditary diffuse gastric cancer (HDGC) [34,35]. HER2 (*ERBB2*) overexpression or gene amplification is observed in 10–30% of gastric cancer cases, leading to a more aggressive disease that is difficult to manage [36,37]. Furthermore, mutations in *TP53*, a crucial tumor suppressor, disrupt apoptosis and elevate VEGF-A production, promoting angiogenesis and accelerating tumor growth and invasion [38]. In hepatocellular carcinoma (HCC), multiple mutations and dysregulated signaling pathways, including WNT, MAPK, mTOR, TGF-β, STAT, and TERT, drive tumor initiation and progression. Mutations in *CTNNB1*, which encodes β-catenin, activate the WNT signaling pathway, leading to increased expression of β-catenin, c-Myc, and cyclins, thereby promoting tumor proliferation, migration, and resistance to apoptosis [39].

In pancreatic cancer, *KRAS* mutations (88%) drive tumor initiation by activating uncontrolled MAPK and PI3K pathways, promoting cell growth [40,41,42]. *CDKN2A* mutations disrupt cell cycle control, allowing unchecked proliferation [43]. *TP53* mutations impair apoptosis and DNA repair, enabling tumor survival [44]. *SMAD4* and *TGFBR2* mutations inactivate TGF-β signaling, reducing growth suppression and enhancing metastasis in pancreatic cancer [45]. In colorectal cancer (CRC), *BRAF* mutations, such as V600E, activate the MAPK pathway, causing uncontrolled cell growth, while CpG island methylator phenotype (CIMP)-related DNA methylation silences *MLH1*, leading to microsatellite instability-high (MSI-H) and defective DNA repair [46]. Loss of *APC* prevents β-catenin degradation, leading to continuous WNT signaling activation, which promotes uncontrolled proliferation, disrupts cell adhesion, and drives tumor formation [47]. Many of the dysregulated pathways described above are directly modulated by carvacrol, making it a promising candidate for targeted intervention. The following sections discuss carvacrol’s effects on individual GI cancers.

## 5. Therapeutic Effects of Carvacrol on Gastrointestinal Cancers

### 5.1. Esophageal Cancer

Esophageal cancer is more prevalent in men and is ranked as the eighth most common cancer worldwide. In 2020, there were an estimated 0.6 million new cases of esophageal cancer, and 0.54 million deaths occurred as a result of this disease, based on GLOBOCAN 2020 [48]. It accounted for 17,650 new cases in the United States and 16,080 deaths in 2019 [49]. The prevalence of this cancer varies significantly across different regions. However, advancements in medical science have improved the five-year survival rate [50,51,52,53]. A study conducted by Lin and colleagues on human esophageal squamous carcinoma KYSE-150 cells showed that carvacrol inhibited cell proliferation in a dose- and time-dependent manner. It also induced apoptosis by activating caspase-3 and caspase-9 while downregulating Bcl-2 mRNA levels, further supporting the occurrence of apoptosis [54].

### 5.2. Gastric Cancer

With 1.1 million new cases and 770,000 fatalities recorded in 2020, gastric cancer ranks sixth in terms of frequency of diagnosis and is a major cause of cancer-related deaths globally. Men are twice as likely as women to get the condition, and projections indicate that cases will rise by 62% to 1.77 million by 2040. While North America and some regions of Africa have a lower incidence, Eastern Asia, Eastern Europe, and South America have the highest prevalence [55,56]. According to studies, carvacrol causes oxidative stress by increasing the production of reactive oxygen species (ROS) and depleting GSH levels, which results in strong lethal effects on AGS gastric cancer cells in a dose-dependent manner (IC_50_ = 82.57 ± 5.58 μmol/L). Apoptosis is caused by this oxidative imbalance, as seen by the development of apoptotic bodies, chromatin condensation, and nuclear shrinkage. Additionally, carvacrol promotes apoptosis by downregulating anti-apoptotic Bcl-2 and upregulating pro-apoptotic proteins (Bax, caspase-3, and caspase-9) [57]. Furthermore, carvacrol exhibits selective cytotoxicity, significantly reducing the viability of AGS cells while having little effect on healthy WS1 cells. Carvacrol’s biological significance is further supported by in vivo investigations conducted on Wistar rats, which show that it causes oxidative stress and gastrointestinal disease [58]. The molecule has dose-dependent effects; at low concentrations (10–25 mg/kg Body weight (BW)), it has anti-inflammatory, antioxidant, and chemopreventive qualities; at higher doses (50–100 mg/kg BW), it acts as a pro-oxidant, enhancing inflammation, oxidative stress, and apoptosis. Furthermore, reduced VEGF expression is linked to high doses, indicating that carvacrol may have a function in preventing angiogenesis in stomach cancers. These results highlight the potential of carvacrol as a natural substance with therapeutic and preventative benefits against gastric cancer [59].

### 5.3. Pancreatic Cancer

**Figure 1:**
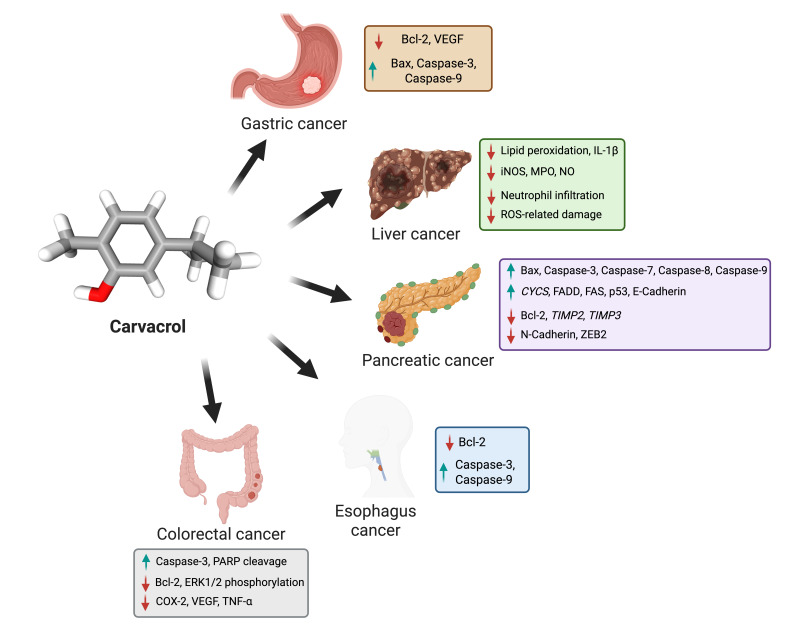
This figure illustrates the anticancer effects of carvacrol on various gastrointestinal cancers, including gastric, liver, pancreatic, esophagus, and colorectal cancers. It highlights carvacrol’s ability to induce apoptosis (via caspase activation), inhibit angiogenesis (by reducing VEGF), suppress inflammation, and decrease oxidative stress. The molecular pathways affected by carvacrol include Bax, Bcl-2, Caspases, COX-2, and VEGF, demonstrating its potential as a natural anticancer agent. The image was created with BioRender.com.

Pancreatic cancer is the 12th most common malignancy globally and the 7th leading cause of cancer-related mortality, with 508,533 new cases and 505,752 deaths reported in 2021 [60,61]. The global age-standardized incidence rate (ASR) is 4.8 per 100,000, with a male-to-female ratio of 1.4:1.0 [62]. It primarily affects the elderly; 90% of newly diagnosed cases occur in people over 55, most frequently in those between the ages of 70 and 80 [63,64]. Akpınar et al. found that carvacrol reduces proliferation, causes apoptosis, and decreases metastasis, indicating its strong anticancer potential in pancreatic cancer, particularly in PANC-1 cells [59]. Its effectiveness is both dose- and time-dependent, as evidenced by the significant reduction in cell viability at 300 and 400 μM after 24 and 48 h. These doses also inhibit carcinogenic mediators such as Atg16L1 and Beclin-1 at the protein and gene levels, which reduces autophagy and migration [65]. Carvacrol triggers apoptosis by altering important apoptotic genes, which shifts the balance in favor of programmed cell death by upregulating pro-apoptotic markers such as Bax, caspase-3, caspase-7, caspase-8, caspase-9, cytochrome C, Fas, Fas-associated death domain (FADD), and p53 and downregulating anti-apoptotic Bcl-2. Furthermore, it is essential for preventing metastasis by limiting the migration and invasion of cancer cells by upregulating epithelial markers like E-Cadherin and tissue inhibitors of metalloproteinases 2 and 3 (*TIMP2* and *TIMP3*) and downregulating mesenchymal markers like N-Cadherin and ZEB2. Because carvacrol has potent anti-proliferative, anti-migratory, and pro-apoptotic properties (Figure 1), it shows promise as a natural treatment for pancreatic cancer [65].

### 5.4. Liver Cancer

Liver cancer is a primary global health concern, ranking as the sixth most commonly diagnosed cancer and the third leading cause of cancer-related mortality. HCC is the predominant histological subtype, accounting for 80–85% of cases, followed by intrahepatic cholangiocarcinoma (CCA) [66,67]. In 2020, there were approximately 905,677 new liver cancer cases worldwide, reflecting a 21.1% increase since 2008, from 748,000 cases, while mortality reached 830,180 deaths, marking a 19.5% rise since 2008, when there were 695,000 cases. The disease disproportionately affects males, ranking as the fifth most diagnosed cancer among men and the ninth among women. Given its increasing incidence and high mortality rate, liver cancer remains a critical focus for global cancer research and therapeutic advancements [68,69]. Carvacrol exhibits potent anticancer effects against liver cancer through multiple mechanisms, including hepatoprotection, apoptosis induction, tumor proliferation inhibition, and drug efficacy enhancement. A study conducted by Jayakumar et al. shows that carvacrol protects the liver from diethylnitrosamine (DEN)-induced hepatocellular carcinogenesis by reducing lipid peroxidation, restoring key liver enzymes (AST, ALT, ALP, LDH, cGT), and enhancing antioxidant defenses (SOD, CAT, GPx, GR, GSH) while selectively inducing apoptosis in cancer cells without harming normal liver tissue [70]. It also inhibits HepG2 cancer cell growth by activating caspase-3, promoting PARP cleavage, downregulating Bcl-2, and modulating the MAPK signaling pathway by selectively reducing ERK1/2 phosphorylation while activating p38 [71]. A study on adult male Sprague Dawley rats (aged 6–8 weeks) demonstrated that carvacrol enhances sorafenib efficacy in HCC, improving survival rates, reducing tumor progression, and mitigating sorafenib-induced cardiac and hepatic toxicity. It also overcomes drug resistance by downregulating *ABCG2*, *NOTCH1*, *SALL4*, and *CD133*. Overall, it shows anticancer effects by decreasing cyclin D1 and Bcl-2 while upregulating Bax and caspase-3, promoting apoptosis, and modulating TRPM7 [72]. Additionally, carvacrol reduces serum alpha-fetoprotein (AFP) and alpha-L-fucosidase (AFU) levels by downregulating COX-2 and oxidative stress, inhibits angiogenesis via VEGF suppression, and prevents tumor proliferation by downregulating proliferating cell nuclear antigen (PCNA) and Ki-67 through TNF-α suppression. It also induces apoptosis through the p53-dependent pathway by upregulating pro-apoptotic genes (Bax, caspase-3, -6, and -9) and downregulating anti-apoptotic Bcl-2, shifting the balance toward programmed cell death in a dose-dependent manner. Collectively, these findings highlight carvacrol (Figure 1) as a promising multi-targeted natural therapeutic agent for liver cancer treatment and prevention.

### 5.5. Colorectal Cancer

CRC is the third most common cancer globally, with 1,926,425 new cases in 2022 and 930,000 deaths in 2020 out of 1.9 million cases [73]. Its burden is expected to rise significantly, reaching 3.2 million cases and 1.6 million deaths by 2040, especially in countries with a high human development index (HDI) score. Lifestyle factors, aging, and diet contribute to this increase, highlighting the need for better prevention, early detection, and improved treatments to mitigate its global impact [74,75]. Carvacrol exhibits significant anticancer and chemopreventive potential against CRC through multiple mechanisms. It reduces cell viability and migration in CoCl_2_-induced hypoxic SW480 cells in a dose-dependent manner (IC_50_ ≈ 324 μg/mL), suggesting its potential as an adjuvant therapy for metastatic CRC [76]. Carvacrol (100 μg/mL) decreased growth in HT-29 and HCT-116 CRC cells, with HT-29 being more sensitive [77]. In gastro studies, carvacrol (40 mg/kg b.wt) significantly reduces tumor burden, inhibiting 92.45% of polyps and aberrant crypt foci in 1,2-dimethylhydrazine (DMH)-induced carcinogenesis, highlighting its anti-proliferative effects and potential as a dietary phytochemical for CRC prevention [23]. Furthermore, carvacrol exhibits gastroprotective effects in DMH/DSS-induced colitis-associated CRC, inhibiting aberrant crypt foci growth, reducing inflammation, and preventing oxidative stress. It lowers lipid peroxidation, IL-1β, inducible nitric oxide synthase (iNOS), myeloperoxidase (MPO), and nitric oxide (NO) levels, suppressing neutrophil infiltration and ROS-related damage, highlighting its potential as a dietary therapeutic agent for inflammation-associated CRC prevention [78].

## 6. Toxicological Profile and Safety Assessment of Carvacrol

Given its potential for therapeutic application, it is critical to assess carvacrol’s safety profile and tolerability, particularly in preclinical settings. Carvacrol, a natural compound commonly used as a food preservative, flavoring agent, and antimicrobial agent, has been extensively studied for its safety and potential therapeutic applications (Table 1). The FDA has approved carvacrol as a safe food additive in the United States and Europe [79,80]. Furthermore, it has been approved as a preservative, successfully limiting the growth of spoilage bacteria and foodborne pathogens while preserving product quality. Several studies have assessed its safety profile, emphasizing its low toxicity and selective effect on malignant and pathogenic cells while sparing healthy tissues. Llana-Ruiz-Cabello et al. conducted an in vivo genotoxic study with doses ranging from 81 to 810 mg/kg BW and found no signs of oxidative stress, gene damage, or DNA toxicity, confirming its safety within this range [81].

Barnwal et al. investigated the preventive effects of carvacrol (25 and 50 mg/kg) against benzo(a)pyrene-induced lung damage in Swiss albino mice [82]. They found that carvacrol pretreatment decreased lipid peroxidation and increased antioxidant enzyme activity, suggesting that it may help reduce inflammation and oxidative stress [82]. According to separate studies, carvacrol (50 and 100 mg/kg) improved antioxidant indicators, liver function, matrix metalloproteinase activity, and histological integrity, protecting against alcohol-induced liver toxicity and avoiding cirrhosis, fibrosis, and steatosis [83,84]. Ghorani et al. investigated the effects of doses of 1–2 mg/kg/day in healthy individuals, and they observed changes in blood parameters, including decreased hemoglobin and HDL cholesterol, as well as increased creatinine phosphokinase [85]. Baranauskaite et al. reported carvacrol’s anticancer activity, showing 50% inhibition of cancer cells at 199–322 μM, confirming its anti-proliferative effects in oregano extracts [86]. Bouhtit et al. show that carvacrol, especially when combined with thymol, effectively eliminates leukemia cells (KG1, HL60, and 70% of K562-resistant cells). However, while the treatment is toxic to normal cells, over 50% remain viable, suggesting selective anticancer effects with low toxicity to healthy cells [87]. At a low dose (10 mg/kg), carvacrol showed protective effects against L-arginine-induced pancreatitis by reducing serum α-amylase and lipase activities, preventing oxidative damage, and minimizing tissue inflammation. However, higher doses (100 and 500 mg/kg) caused pancreatic damage, increasing α-amylase levels, inflammatory cell infiltration, and edema, indicating a dose-dependent effect [88]. In contrast to PANC-1 cells, human non-tumorigenic pancreatic epithelial cells (HPDE) were unaffected by carvacrol treatment. This suggests that carvacrol exhibits selective cytotoxicity, targeting cancerous cells while sparing normal cells.

## 7. Role of Nanotechnology and Synergism

Despite carvacrol’s promising effects in vitro and in vivo, limitations such as bioavailability and solubility challenge its therapeutic application. Emerging strategies in nanotechnology and drug synergism aim to overcome these hurdles.

### 7.1. Nanotechnology Applications

As a monoterpene phenolic compound with strong anticancer effects, carvacrol faces several obstacles to clinical use, including high volatility, poor solubility, and limited bioavailability. By increasing carvacrol’s stability, extending its shelf life, and permitting controlled release, nanotechnology presents a promising solution that boosts the drug’s therapeutic efficacy [89,90]. Furthermore, by functionalizing them with particular ligands, nanoparticles can more efficiently target and bind to cancer cells. When natural compounds and nanotechnology are combined, drug availability, distribution, and tumor targeting significantly increase, resulting in safer and more effective treatments [91]. Nanotechnology significantly improves carvacrol’s anticancer potential by addressing solubility issues and encouraging selective accumulation at tumor sites [92]. Carvacrol nanoparticles significantly increase anticancer efficacy, especially when combined with doxorubicin. They enable targeted delivery, lower resistance, and require less dosage, increasing the active compound’s potency and stability [93].

In contrast to single drug-loaded nanoparticles, Maryam et al. demonstrated that combining carvacrol and chemotherapy agents in HSA nanoparticles may provide a more effective strategy for treating gastric cancer by enhancing anticancer efficacy and possibly lowering side effects [94]. Carvacrol’s stability and bioavailability are enhanced by the Tin oxide-sodium alginate-polyethylene glycol-carvacrol (SnO2-SAPG-Carvacrol) nanocomposite, which also exhibits potent anticancer effects against esophageal squamous carcinoma KYSE-150-7 cells. It causes oxidative stress, raises ROS, alters the potential of the mitochondrial membrane, and lowers GSH and SOD levels, all of which cause cancer cells to die. Additionally, the nanocomposite suppresses migration, downregulates cyclin D1, and increases pro-apoptotic proteins and caspase activity to promote apoptosis [95].

### 7.2. Synergistic Therapeutic Approaches

Carvacrol could also be employed with existing chemotherapeutics for a synergistic effect. Interestingly, studies have demonstrated a synergistic effect between thymol and carvacrol in leukemia treatment. Research by Bouhtit et al. reported that while the K562 cell line exhibited reduced sensitivity to individual therapies, the combination of thymol and carvacrol resulted in a 70% reduction in cancer cells with IC_50_ values ranging from 0.92 to 1.70 µg/mL [87]. In a separate report, Taibi et al. discovered that the thymol-carvacrol combination had strong cytotoxic effects, indicating higher bioactivity at lower concentrations [96]. Another study by Azimi et al. reported that combining carvacrol with 5-fluorouracil (5-FU) considerably enhanced the proportion of apoptotic cells compared to treatments with either drug alone, highlighting its potential as a synergistic anticancer therapy [97].

## 8. Conclusions and Future Perspectives

Carvacrol, a bioactive monoterpenoid compound derived from various aromatic plants, demonstrates promising anticancer properties across multiple GI malignancies. By modulating key molecular pathways involved in apoptosis, angiogenesis, metastasis, and inflammation, it supports its potential as a complementary therapeutic agent. Nanotechnology-driven formulations and synergistic combinations with established chemotherapeutics further enhance its potential clinical relevance.

Despite substantial preclinical evidence supporting carvacrol’s anticancer efficacy in GI malignancies, its clinical application remains underexplored. To bridge this translational gap, a multi-faceted approach is essential. Foremost, early-phase clinical trials are needed to evaluate carvacrol’s safety, tolerability, and therapeutic window in human subjects. Concurrently, pharmacokinetic profiling and dose-optimization studies should be conducted to improve its systemic availability and therapeutic consistency. Advanced formulation strategies, particularly nanocarriers-based delivery systems, can further enhance tumor-specific targeting while minimizing off-target toxicity.

An important avenue for future investigation is the integration of carvacrol into combination therapy regimens. Co-administration with existing chemotherapeutics or immunotherapies may prevent adverse drug interactions or potentiation of side effects. In parallel, incorporating carvacrol into a precision oncology framework will require the identification of predictive biomarkers and molecular signatures that can guide patient selection and maximize therapeutic benefit.

Ultimately, realizing carvacrol’s full potential in GI oncology will demand robust interdisciplinary collaboration spanning pharmacology, molecular oncology, clinical research, and natural product chemistry. Key challenges, such as variability in patient response, bioavailability limitations, and formulation standardization, must be systematically addressed. With sustained research efforts and clinical validation, carvacrol holds significant promise as a low-toxicity, multi-targeted therapeutic agent for the prevention and treatment of GI cancers.

## Figures and Tables

**Table 1:** Overview of the mechanistic insight of carvacrol in gastrointestinal cancers. ↑ indicates upregulation; ↓ indicates downregulation.

Serial number	Type of Cancer	Biological Model	Physiological Effects	Mechanism of Action	References
1	Esophageal cancer	KYSE-150	Inhibits cell proliferation and induces cell apoptosis.	↑ caspase-3 and caspase-9 and ↓ Bcl-2	[54]
2	Gastric cancer	AGS	Inhibits cell proliferation-induced DNA damage, apoptosis, and ROS generation.	↓ Bcl-2 and ↑ Bax, caspase-9, and caspase-3	[58]
3	Pancreatic cancer	PANC-1 cells	Triggers apoptosis while demonstrating anti-proliferative and anti-migratory properties.	↑ epithelial markers like E-Cadherin, TIMP2, TIMP3, Bax, caspase-3, caspase-7, caspase-8, caspase-9, cytochrome c, FADD, FAS, and p53 ↓ mesenchymal markers like N-Cadherin, ZEB2, and Bcl-2	[65]
		PANC-1 cells	Inhibits proliferation, induces apoptosis, and suppresses metastasis.	Inhibit Atg16L1 and Beclin-1	
4	Liver cancer	HepG2	Hepatoprotection, inducing apoptosis and inhibiting tumor proliferation.	↓ lipid peroxidation, restoring key liver enzymes (AST, ALT, ALP, LDH, cGT) and ↑ SOD, CAT, GPx, GR, GSH	[70]
		HepG2	Causes apoptosis.	↑ caspase-3, promoting PARP cleavage, ↓ Bcl-2, and modulating the MAPK signaling pathway ↓ ERK1/2 phosphorylation while activating p38	[71]
5	Colorectal cancer	SW480 cells	Anti-proliferative and inhibiting angiogenesis.	↓ TGF-α and ↑ anti-growth factors VE	[77]

## Data Availability

No new data were created or analyzed in this study. Data sharing is not applicable to this article.
